# Supervision of community peer counsellors for infant feeding in South Africa: an exploratory qualitative study

**DOI:** 10.1186/1478-4491-8-6

**Published:** 2010-03-30

**Authors:** Karen Daniels, Barni Nor, Debra Jackson, Eva-Charlotte Ekström, Tanya Doherty

**Affiliations:** 1 Health Systems Research Unit, Medical Research Council, South Africa; 2 Nordic School of Public Health, Sweden; 3 Department of Women's and Children's Health, Uppsala University, Sweden; 4 School of Public Health, University of the Western Cape, South Africa

## Abstract

**Background:**

Recent years have seen a re-emergence of community health worker (CHW) interventions, especially in relation to HIV care, and in increasing coverage of child health interventions. Such programmes can be particularly appealing in the face of human resource shortages and fragmented health systems. However, do we know enough about how these interventions function in order to support the investment? While research based on strong quantitative study designs such as randomised controlled trials increasingly document their impact, there has been less empirical analysis of the internal mechanisms through which CHW interventions succeed or fail. Qualitative process evaluations can help fill this gap.

**Methods:**

This qualitative paper reports on the experience of three CHW supervisors who were responsible for supporting infant feeding peer counsellors. The intervention took place in three diverse settings in South Africa. Each setting employed one CHW supervisor, each of whom was individually interviewed for this study. The study forms part of the process evaluation of a large-scale randomized controlled trial of infant feeding peer counselling support.

**Results:**

Our findings highlight the complexities of supervising and supporting CHWs. In order to facilitate effective infant feeding peer counselling, supervisors in this study had to move beyond mere technical management of the intervention to broader people management. While their capacity to achieve this was based on their own prior experience, it was enhanced through being supported themselves. In turn, resource limitations and concerns over safety and being in a rural setting were raised as some of the challenges to supervision. Adding to the complexity was the issue of HIV. Supervisors not only had to support CHWs in their attempts to offer peer counselling to mothers who were potentially HIV positive, but they also had to deal with supporting HIV-positive peer counsellors.

**Conclusions:**

This study highlights the need to pay attention to the experiences of supervisors so as to better understand the components of supervision in the field. Such understanding can enhance future policy making, planning and implementation of peer community health worker programmes.

## Introduction

### Supervising community health workers

Community or lay health workers have been defined as "any health worker carrying out functions related to health care delivery; trained in some way in the context of the intervention, and having no formal professional or paraprofessional certificate or degree in tertiary education" [1]. The concept of community health workers (CHWs) has been around for at least 50 years [2]. In particular, they were strongly promoted in the years following the International Conference on Primary Health Care at Alma-Ata (1978) [3,4]. Then they were seen as a means to improving primary health care in developing countries [4-6] and reaching the goal of Health for All by the year 2000 [3]. The 1980s thus saw a flurry of such programmes [4-6], but subsequent failure to produce the expected outcomes led to a decline in enthusiasm for national community health worker programmes [4,7]. Recent years have however seen a re-emergence of such programmes [2], especially in relation to HIV care [4], and in increasing coverage of child health interventions [7]. In the face of human resource shortages and fragmented health systems, many countries are investing in CHW programmes in an attempt to achieve the goal of child survival. There are an array of child health interventions in which CHWs partake, including health promotion, disease prevention and more complex interventions such as prevention of mother-to-child HIV transmission [7]. The WHO has placed particular emphasis on the role of community support in its Global Strategy for Infant and Young Child Feeding [8]. Within this strategy, CHWs functioning as peer supporters or counsellors are strongly encouraged.

Given the now long history of CHWs, much has been written about the impact such programmes can and do have, why some of these programmes have failed, and what can be done to strengthen existing and future programmes [2]. Within all of this discussion, the role of supervision has been key [6]. It has been argued that continuous and educative supervision is indispensable to CHW activities[3]. For example the National Institute of Health and Clinical Excellence in the United Kingdom strongly recommends supervision as a key strategy in recruiting and retaining infant feeding peer supporters and lay counsellors [9]. The strength or weakness of the supervision CHWs receive has also been linked to the level of the quality of care they are able to deliver [6]. Regular supervision is particularly important for rural CHWs who may otherwise feel isolated [3]. However, supervision has been shown to be a persistent weakness in CHW programmes [6], with suggestions of infrequency and inadequacy [3]. While good supervision requires sufficient funding [6], the balance of spending in interventions may not always favour supervision [10]. Supervision exists on a continuum with higher level supervisors supporting frontline supervisors [11]. The responsibility for supervision, however, has been shifted downwards without adequate support [12]. Thus, while supervision is identified as the vehicle for assuring quality health services, "typically it does not receive the support--human or financial--required to fully carry out and sustain supervisory activities" [12].

While offering valuable insights, much of what has been written about CHW supervision has been anecdotal. The strength of the methods of those few studies that there are has been questioned [13]. Furthermore, the evidence is not unequivocal and thus some findings have contradicted each other. For example, a study conducted in Nepal (1995) showed that CHWs performed better after receiving increased training, supervision and supplies [5]. Conversely a more recent study (2007), conducted with CHWs in Kenya, has shown that multiple interventions which included supervision and refresher training were ineffective as quality improvement strategies [13]. There is therefore room for more accurate documentation and description of supervisory activities, and their relationship to intervention success or indeed failure. Based on our reading of the literature, the voice of the supervisors themselves is strikingly missing. This paper therefore addresses this gap through describing the experiences of supervisors by their own account. It is based on a set of qualitative individual interviews with each of the three supervisors employed as part of a CHW peer counselling intervention to promote appropriate infant feeding practices. The paper aims to present the findings from these interviews describing the supervisors' tasks by their own account and the facilitating factors and challenges they faced in carrying out these tasks.

### A CHW intervention study in support of infant feeding in South Africa (the Promise EBF study)

Infant feeding in South Africa, especially in the context of HIV, poses particular health, social and structural challenges [14-18]. While vertical transmission of HIV from mother to child is a risk during breastfeeding [18], this risk is increased when the mother feeds the child with both formula milk and breast milk (locally referred to as 'mixed feeding') [17,19,20]. However there appears to be little difference in HIV-free survival between exclusive formula feeding and exclusive breastfeeding, due to the fact that the HIV infections through breastfeeding and the infectious disease mortality through formula feeding balance each other out [17,21]. Therefore, in South Africa, HIV-positive mothers are advised to choose between exclusive formula feeding and exclusive breastfeeding. If they choose exclusive formula feeding then the public health service provides formula milk to the mother at no cost for the first six months. However, studies have shown that it is very difficult for mothers to sustain exclusive infant feeding with either formula or breast milk [14-16]. Mothers face structural challenges such as poor counselling from health professionals and an inadequate supply of formula; economic pressures such as having to change feeding practices (from breast to formula) when returning to work; and social pressures such as being exposed and stigmatised as HIV positive if found to be exclusively formula feeding [14-16]. Community based peer counselling in low and middle income countries has, however, been shown to be effective in assisting mothers in maintaining a choice of exclusive breastfeeding [22-26]. Drawing on the success and lessons from these studies, the Promise EBF community based randomised controlled trial (RCT) utilising CHWs trained in infant feeding peer counselling was initiated (Clinicaltrials.gov: NCT00397150). The trial is being conducted in Zambia, Burkina Faso, Uganda and South Africa.

The RCT in South Africa is based in three investigation sites with high HIV prevalence [27]. The sites, although all characterised by poor socio-economic conditions, were very different from each other. Paarl is a peri-urban/rural site situated in an area of commercial farming. The Infant Mortality Rate (IMR) is around 40/1000 live births. This site has an average of 289 new antenatal bookings per month. The HIV prevalence amongst antenatal clients is 9%. Rietvlei (Umzimkulu sub-district) is in one of the poorest rural areas of South Africa, with an IMR of 99/1000 live births. The hospital has an antenatal clinic and delivers approximately 170 women per month. The antenatal HIV positive rate is 28%. Umlazi is a peri-urban settlement close to Durban that has a mixture of formal and informal housing. The IMR is around 60/1000 live births. On average 248 women book for antenatal care each month. The HIV prevalence amongst antenatal clients is 44% [28].

The RCT investigation in South Africa aims to "determine the effect of community peer counsellors on rates of exclusive infant feeding (i.e. exclusive breastfeeding and exclusive formula feeding)" [27]. In the intervention arm, mothers received infant feeding peer counselling, while in the control arm peer counsellors assisted mothers in accessing social support grants. This qualitative sub-study forms part of the process evaluation of the larger RCT, with the intention that insights gained here will enhance understanding of the intervention process.

Currently in South Africa, mothers receive infant feeding counselling from health professionals through the public health system. However, this counselling has been shown to be insufficient [29]. This intervention therefore has been designed to provide mothers with additional support at a community level. It employed local women to provide community peer counselling on infant feeding to mothers in their villages or townships. The peer counsellors were selected on the basis of their educational level, their commitment to community development and infant feeding, and their counselling skills. Prior breastfeeding experience was not a requirement. Peer counsellors were trained for two weeks: one week in the class and one week in postnatal wards. The content of the course included benefits of exclusive breastfeeding, dangers of mixed-feeding, safe preparation and storage of infant formula, breastfeeding management, management of common infant illnesses, counselling techniques, how to encourage women to know their HIV status and how to support women to disclose their HIV status to their immediate family and/or partner [30]. Thereafter, peer counsellors were assigned the task of recruiting pregnant women in their villages or townships to participate in the study. If the women consented, then they would receive one antenatal support visit, followed by postnatal support visits in weeks 1, 4, 7 and 10. These visits were intended to support whatever choice the mother had made with the health professional, rather than to influence her choice.

The delivery of the intervention was managed at each site by a peer counsellor supervisor who was situated at a local intervention office. Three well-performing staff from a prior research project at the same three study sites were promoted to become peer support supervisors. Each of these three supervisors managed and supported between 10 and 12 peer counsellors. The role of the supervisor was to provide support to these peer counsellors and to encourage high quality consistent counselling[28]. There were monthly face-to-face meetings with the supervisors and peer counsellors where the peer counsellors submitted their visit forms and had a discussion with the supervisors about any problems they faced in the previous month. The supervisor had at least one contact session (telephonically or face-to-face) with each peer counsellor each week and observed the counselling of each peer counsellor during a home visit at least once a month. The supervisors received the same intervention content training as the peer counsellors with additional attention to the roles and tasks involved in the supervision process. The supervisor and peer counselling training was developed by members of the research team in communication with principal investigators of previous breastfeeding peer counselling studies [31]. Supervisors were supported telephonically and in person by a junior member of the research team who liaised directly with senior research staff.

### Ethical approval

This study received ethical approval from the University of the Western Cape. Each respondent was asked in advance (verbally and through email) if they would be willing to be interviewed. On the day of the interview the interviewing process and the informed consent forms were explained to respondents. Each agreed to participate and signed the consent forms.

## Methods

This qualitative study was conducted between July and August 2006 in the three study sites of the Promise EBF trial in South Africa. The peer counselling supervisor of each site was interviewed individually by the first author in a private setting at or nearby the intervention study office. The interviews were conducted in English, a language in which each of the supervisors is fluent, although though it is not a first language for any of them. Two of the interviews were just over an hour long and the third lasted 45 minutes. The interview schedule was discussed in advance between the first author and the second author. Each supervisor was asked about their background prior to this intervention and then about their experience within the intervention. A request to do the interview along with information about the nature of the interview was sent to each supervisor in advance of the visit to their site. While each of them agreed to be interviewed and signed the informed consent forms before the interview, it is likely that none of them felt that they had the choice to refuse to be interviewed, given that the research was being conducted by their current employers. Furthermore their absolute anonymity in the reporting process could not be guaranteed especially since there were only three supervisors in the intervention. A concern before the interviews therefore was that the supervisors would feel compelled to present a positive view of their experiences and thus that the interviews may be biased. However, despite the lack of anonymity, each of the supervisors offered very frank observations, describing both positive and negative experiences. The interviewer also practised reflexivity through the interviewing process by taking note of how she may have influenced the interviewees' responses.

The interviews were recorded using electronic audio-recording equipment. These electronic files were transcribed verbatim by a transcribing service and checked by the first author. These transcripts formed the basis of the qualitative data analysis. The analysis commenced with the first and the second author reading and annotating each transcript individually. They then met over two weeks during which they discussed their impressions of these transcripts and other interview data. During this time they agreed on an overall framework or model through which the interview data could be described. Following this meeting the first author categorised the text of the interviews based on this agreed framework, adapting the categories as necessary. The document containing the categorised quotations was then shared and discussed with the second and last author. Following this discussion, the results were written up and shared with the broader research team for further validation. The key categories distilled out of this process are presented and described here as findings.

## Results

Beyond a description of the tasks, facilitators and challenges of supervision, the interviews showed the uniqueness of each supervisor. Through these interviews we could see how personality, background and context influenced experience of the intervention and how the intervention process is shaped through each individual supervisor's understanding of the supervisor role. Thus, below we present a vignette on each supervisor before discussing the interview data.

### Describing the supervisors

#### Supervisor A

The youngest of the three supervisors was based in the township adjacent to an agricultural town. She was born and raised there and showed sensitivity to the cultural nuances of her community. Her background included active involvement in her church and undergraduate study in counselling and psychology. Her approach to supervision was largely that of being an open and available support to the peer counsellors, especially in relation to what she perceived as the emotional vulnerability embedded in the task.

#### Supervisor B

This supervisor was based in a rural setting. In general, the setting was resource poor and supervision required travelling vast distances, often on dangerous roads. Of the three supervisors she felt least supported. She started in this project as a research assistant before which her employment was largely clerical and administrative. This was reflected in her approach to supervision, which was largely that of administering the intervention and ensuring the completion of tasks.

#### Supervisor C

This supervisor was based in the township near to a large city. She described herself as an "old girl". The oldest of the supervisors, she was the only one with a professional health background, having been a neonatal nurse and midwife. She had however left the practice of nursing many years ago and had since been working with a variety of research projects. Of the three supervisors, her approach was both the most managerial and the most technical. She was also the only one to have considered the implications of the intervention for the broader health system.

### Description of the interview data

#### People management: fulfilling the task of supervising peer counsellors

While each of the supervisors offered very different descriptions of their work life and their day to day tasks, across all three interviews it was clear that their primary task was that of people management. This task comprised several facets, each being given different priority depending on the supervisor. Although ensuring the technical soundness of the delivery of the intervention featured strongly in their descriptions, they also described a range of other tasks which were closer to support than technical supervision. These included mentoring and motivating staff; managing the administrative, emotional, and safety demands of the project, setting boundaries for the peer counsellors and acting as an interface between the peer counsellors and the research management team.

##### Administrative management

As to be expected in the implementation of a large intervention study, supervisors were required to do some administrative tasks, including checking the peer counsellors records and financial management. Two of the supervisors also suggested that they were involved in some oversight of the data collection process but this was not strictly within their assigned tasks.

##### Technical oversight and training

The supervisors stressed the importance of technical soundness in the delivery of the intervention. For them, mothers needed to be shown how to feed their infants correctly.

When you're having a counselling session, if a mother has chosen to breast feed then you have to show her how to do it, the positioning and the attachment, all of those things. (A)

Assuring the peer counsellors' technical competence through training and continuous observation in the field was therefore a key component of the supervisors' task. This required an attitude of attentiveness in recognising the extent of the peer counsellors' knowledge. It also required the supervisors to be with the peer counsellors in the field so as to immediately check and correct what they were doing in practice.

*For most of the peer counsellors, it was their first time employment, to ever get a job in their life... some of the terms used in the training were new to them, they wouldn't really understand them properly when they were talking to the mothers. At that time I came in and I did visits with them*... (C)

*Telephone call support it's not effective at all for myself because the peer supporter only tells you what she thinks you need to know but you haven't seen what she did and that's the difference. But when you're there you are able really to give the support that she needs because you've seen what she was doing and you see what she needed to do and you also see where she can improve what she could have done*. (A)

##### Emotional support

One supervisor in particular drew attention to the intervention being emotionally demanding on the peer counsellors (A). Overall she felt that entering into mothers' homes made peer counsellors vulnerable, and therefore they needed the supervisor's support:

*... now someone is basically looking after peer counsellors, because when you go into someone's home you don't know what to expect and how is that going to touch your life*... (A)

Specifically, she described helping peer counsellors in dealing with the frustrations of mothers not adhering to their advice and the difficulties of not being able to intervene in instances where they perceived poor parenting on the part of their clients. The intervention management team (including the supervisors) therefore responded by offering self-care workshops aimed at assisting peer counsellors to cope with these emotional demands:

*I've experienced a lot of time when they just felt so overwhelmed, ... that's how the "self-care" workshop came about ... It helped a lot because they sat as a group not as individuals, they talked about the challenges that they've met and how they can handle that situation in future*. (A)

Supervisors also engaged in emotional support by setting boundaries in order to protect the peer counsellors. As supervisor A suggested, peer counsellors came face to face with the problems of the households to whom they were delivering the intervention. She argued that this could induce feelings of helplessness in them because they could not do anything about these problems. This was overcome through defining the limits of their task: *"so in a way also trying to protect them saying 'this is how far you can go' "*. (C)

##### Mentoring and motivation

As suggested in a quote above, for several of the peer counsellors this was their first ever formal employment and this took some adjustment. As such, supervisors described the need to mentor and motivate staff, ensuring that each of them understood the intervention and that they acknowledged this as an important job.

*It is a bit of a challenge to work with them, they are old people. Sometimes they come here and report that "no, I didn't manage to recruit because my husband was sick or my mother-in-law was like this"... What I want them to feel is that we are working here. At home *[others]*undermine your job. So you have to say 'I am also working', you have to be proud of your job*. (B)

##### Safety considerations

As a reflection of the South African context in which the intervention was implemented, one of the tasks for supervisors was to ensure that their peer counsellors remained safe. This was particularly important, since peer counsellors travelled on foot to visit mothers who lived in poor socio-economic areas prone to violence and drug abuse.

*The areas are not safe for peer supporters ...we had a peer supporter who went visiting the house and somebody was shot... in her presence... When you in the community there's no way we can separate these things. We live with this kind of life in townships and you just need to be very careful when you there*...

*... I said maybe you should avoid that visit, phone her and ask if you can meet somewhere, or just avoid going there because if you get assaulted we will not be able to handle that, it might just be difficult for us*. (C)

#### Making the job possible: facilitating peer counselling supervision

As a starting point to fulfilling their tasks, supervisors needed to be clear about what their job function was, what potential challenges the peer counsellors might face in the field and what the boundaries of the intervention were. This understanding combined with their work and life experience prior to this intervention shaped their focus. Thus the supervisor who had a nursing and research background focused largely on the technical aspects of the intervention. Likewise the supervisor who displayed the strongest interest in counselling focused much of her discussion on emotionally supporting her staff. One of the supervisor's prior experiences seemed to be limited to that of being an administrator, and in her interview she spoke mostly of her various administrative duties.

Linked to their abilities was the attitude displayed by the supervisors. Two of the supervisors showed a strong sense of authority, self confidence and self awareness. Through this attitude they were able to address issues that arose in the field. As pointed to above they took it upon themselves to organise workshops and training sessions which would address the emotional and information needs of the peer counsellors.

*For me as, as an old researcher I could see a lot of gaps and that really needed me to work very hard in supporting them*. (C)

Throughout their interviews there is a sense that they felt that they were in charge. But this attitude did not mean a sense of disrespect. The role of supervisor was still deeply embedded in the cultural context in which they worked:

*I'm very young to them. I think there are only three or four *[peer counsellors] *that are younger than myself. So being able to know how to address people that are older than you and yet you are the one that is supposed to give support to them and to tell them this is where you need to improve and that you can do better ... I am able to do this and give that element of respect. You always have to have that cultural background, although I'm your supervisor but I always have to give you that kind of respect because culturally *[that's] *how I'm supposed to behave and yet in my work this is what I'm supposed to do*. (A)

As will be described in more detail below, the supervisors also faced challenges related to dealing with their staff being HIV positive, and this attitude of being able to take charge influenced their ability to face these challenges. This attitude however was enhanced by a good relationship with the research management team in which the supervisor herself felt supported. It was also important that she felt that she had the scope within the project to creatively deal with her challenges.

#### Difficulties and challenges

The difficulties and challenges for supervisors in this intervention were largely contextual, but they were also structural, and to some extent linked to the supervisor themselves.

##### HIV

This intervention was implemented in areas of high HIV prevalence. The presence of this disease and the prevailing attitude of secrecy towards it, proved challenging. Not all peer counsellors understood the process of vertical transmission in relation to infant feeding:

*We discovered that there were things that they didn't properly understand like the virus in the milk, some said yes there is some said no*. [A senior researcher] *told me that if the mother is asking them this question when they are counselling, they might just have a problem around that and then we started explaining *[through training]. (C)

Mothers were not required to disclose their HIV status but peer counsellors found it difficult to support them without this knowledge. Supervisors had to help peer counsellors understand that they could not insist on disclosure while at the same time teaching them how to deal with disclosure when it did happen.

*We realised that it's difficult to support when you don't know the status of the mother... but then again your core business is not really to be hunting for HIV-positive people, looking at their symptoms... yours is to support*. (C)

The data also suggested that the process of HIV testing and treatment was problematic and complicated. Since mothers discussed the testing process with the peer counsellors, the supervisors needed to ensure that the peer counsellors were prepared to deal with this:

*Through the peer supporter, we are encouraging people to go and do antenatal care and test. Some mothers ask a lot about this, the results, if they are accurate. Some tell you that the results were really not given to them in a way that it should be done; we know that this should be very confidential, but some mothers don't have that confidentiality. And again there are questions that they comfortably ask you at home about this *[PMTCT drug], *what does it do? 'If I've taken it, should I take it again'*. (C)

The challenge of HIV was however not limited to the mothers being supported but very definitely extended to the peer counsellors themselves. This challenged the supervisors' way of thinking:

*At the beginning when hmm, when I was told about the illness, I said to myself wait a minute, what's going on now, you know? I thought we were peer supporting, now we having the peer counsellors ill. Then I quickly corrected myself that I must not be judgmental, this is a challenge and I mustn't separate them from the community, they are part of the community, what affects this community will also affect them*. (C)

It also challenged supervisors' ability to cope and highlighted a need for them to be supported themselves:

*I sometimes also feel that I need some counselling of some sort, myself, because I sit at home sometimes and think 'Good heavens, she is ill again, what does one do?' *(C)

Sadly, but realistically, supervisors also saw this in relation to the practical challenge of losing staff:

*Because if that happens we need to train more people and training more people needs money and it's time - and sometimes we don't have *[intervention areas] *that's supported because we still have to train you before we take you to the field. So, all those things work on one*. (C)

HIV really shifted supervision out of the confines of delivering the intervention into personal support:

*I do visit the family as well and I still support her ...she tells me she is interested in the job ... as soon as she gets better, she'll be back at work. Hmm, she's not the only one *(C)

##### Rural isolation

The issue of rural isolation pointed to in the literature [3] emerged in our data too. Supervisor B felt strongly that she was left alone to manage a remote rural site with limited interaction with her managers.

*I was doing things on my own ... any problem that is occurring in the office, they looking *[to] *me*. (B)

While the other two supervisors were in close physical proximity to the research management team, this supervisor interacted with management more often telephonically.

##### Staff salaries and attrition

The challenge of staff salaries and attrition was both structural and contextual. Peer counsellors were employed using the same conditions as prescribed for community health workers nationally [27]. Despite salaries being increased during the course of the intervention, supervisors still found themselves dealing with high staff turnover:

*They started last September, that was the first group that came in but because people just found better jobs and then we keep training new people*. (A)

*I'm still experiencing the Department of Health threatening to take these people, promising them ... 'Ah we are going to offer you something, we want you to go for home based care training which after that we will give you salary of 3000' *[ZAR]*'. And then I ended up losing those people*. (B)

Supervisors found it hard to deal with the complaints that they received every month over salaries and one of them suggested that if peer counsellors were paid more they might perform better. More so than in any of the other aspects of the intervention, when it came to the issue of salaries the supervisors were regarded by peer counsellors as the face of and the interface with the research management team.

##### Delivering a research intervention

Although each of the supervisors was employed to support the delivery of the intervention rather than to engage in the research, it was hard for them to avoid the research component. One of them got involved in managing the data collection. One tried not to get involved but offered her assistance. The other found that the data collection took priority:

*Of the challenges that has been there especially for me and that made me to take a back seat, you find that there was a prioritising in the data collection*....

*So you find that there's importance over what the data collectors do and it's a sense of emergency ... I also addressed this with *[the research management team]*this importance *[of] *what the data collectors do over, the supervision, over the peer counsellors*. (A)

While the issues around data collection were specific to this research intervention context, distraction due to other related projects in a site could occur in any context.

## Discussion

The WHO strongly encourages peer counselling as part of community support for infant feeding [8]. The place of CHWs in child survival, including the role of infant feeding peer counsellors, is well argued [7]. Given the burden of poor child health in developing countries [32] and the potential effectiveness of CHWs [33] in the context of human resource shortages, the role of CHWs has become indispensable. Yet as Cattaneo [34] argues, there is a need to look at how this recommendation for peer counselling is put into practice, especially since the recommendation for community support has not been universally successful in practice [35,36]. The question that then arises is: how to optimise CHW effectiveness and how to ensure the best quality of care from such interventions? In this regard, supervision has been cast in the literature as an essential but somewhat weak link in CHW interventions [6]. Unfortunately there is little recent empirical research on what supervisors do, thus offering a limited knowledge base from which to design new policies, programmes and strategies for effective supervision. Our study, though small, begins to fill this gap by listening to the voices of CHW supervisors active in supporting infant feeding peer counsellors.

Both within the literature[11,12] and within current CHW policy in South Africa[37], supervision is primarily discussed in relation to quality assurance. Our data have shown that supervision is about more than simply ensuring the technical competence of peer counsellors in their delivery of the intervention. Throughout the narratives of our three interviews, supervision is equated with support, whether this is technical, emotional or other kinds of support. In trying to clarify what support means in the context of breastfeeding support, Moran et al. [38] turn to a conceptual framework of social support developed by Sarafino[39]. Using this framework they present support as being made up of the following components:

• "Emotional support: the expression of empathy, caring and concern toward the person;

• Esteem support: positive regard for the person, encouragement and agreement with the individual's ideas or feelings;

• Instrumental support: direct assistance of a practical nature;

• Informational support: giving advice, directions, suggestions, or feedback about how the person is doing;

• Network support: provides a feeling of membership in a group of people who share interests and social activities."

Network support, later categorised by Sarafino [40] as Companionship Support, is facilitated by "the availability of others to spend time with the person" .

This conceptual framework can usefully be applied to how our supervisors have described their activities. It is clear from their narratives that they engaged at some point in the course of their duties in each of these kinds of support:

• they supported peer counsellors through the emotional demands of their task;

• they built up the esteem of women who had never previously worked outside of their own homes;

• each of the supervisors engaged in instrumental and informational support through their weekly meetings and field visits;

• these same weekly meetings provided network support through which the group members could learn from each others' struggles.

Supervision thus was not just a management function. Using Sarafino's definition [39,40], supervision can be seen as an extension of the social support peer counsellors offered in the community, now offered to the peer counsellors themselves. This then raises questions around how we define supervision, what we require of supervisors, and how we prepare incumbents for what they will be faced with in the field. The training offered to our supervisors focused largely on the content of the intervention. We have no doubt that this kind of training is standard to many interventions. Yet, when using the framework of social support suggested above, this training is really only preparing incumbents for the tasks of informational and instrumental support. In our data, each supervisor's capacity to offer support beyond this was facilitated by her background and the support she received from her managers--more so, possibly, than by her intervention training. Future programmes could benefit by making explicit the components of support, and ensuring that supervisors are prepared for each aspect.

Beyond the support which our respondents gave to the peer counsellors, their narratives also reveal that they needed to feel structurally and emotionally supported by senior management. They needed to know who to turn to with a problem and they needed to have all the necessary tools for the job, including a clear job description, a proper office and safe transport. In containing the emotional demands which peer counsellors experienced, our supervisors were strengthened by having a senior manager whom they too could turn to.

Overall, each of our supervisors performed to expectations in terms of making contact with the peer counsellors and giving them support. But each of them undertook this in a different way--one focussing more on the administration, another on the intervention and the third on emotional support. This suggests that people do not come into supervisory positions with equal experience and equal skills. The narratives reveal a need for supervisors to have their backgrounds recognised, acknowledged and, where necessary, accommodated for with further skill development. This may ensure that an unequal background does not disadvantage individuals wanting to perform their tasks adequately. The individuality of each supervisor can be nurtured while at the same time building skills to deal with the task at hand.

This intervention posed a new challenge not addressed in the literature: the challenge of peer counselling specifically, and community health work in general, in the context of HIV. These interviews show clearly the impact of HIV on human resources for health. HIV was not just a problem that peer counsellors had to deal with in the community; it was a problem they had to deal with themselves. Whether the intervention is infant feeding, treatment support, immunisation or anything else within the range of services CHWs provide, this problem will persist in areas of high HIV prevalence. It will require careful thinking, careful planning and more than adequate support. Hein Marais so eloquently pointed out that "Most of the burden of AIDS care is being displaced into the invisible zones of the home - and onto the shoulders of women" [41]. How do we ensure that we do not displace the responsibility for HIV care of our CHWs onto the peripheral zone of supervision?

### Key lessons

• There are components of supervision, well beyond technical support, that need to be recognised and prepared for.

• Supervision need not be the weak link in CHW interventions. It can be done well if the supervisors themselves feel supported.

• Supervisors were challenged to contain the difficult context in which peer counsellors had to work, including dealing with poverty and HIV. This raises the question: who supports the supervisor and how this support can be enhanced?

• This study has highlighted the impact of HIV on the CHW experience, and there is still much to be learned in this regard.

### Strengths and limitations

The greatest strength of this study is that it reflects the experience of supervisors by their own account. Conducting individual interviews allowed the interviewer to fully explore each of the respondents' reflections and observations. This has enhanced the depth with which the research question could be explored. This depth has been further enhanced by having the interviews and the analysis conducted by the first author. In this way the first author could fully interact with the data and draw on her own reflections during the research process. All reflections and observations are therefore empirically grounded.

This study may be critiqued for the size of its sample and the fact that it draws on only one intervention, thus raising concern around transferability. Like most qualitative research, this study, however, does not make an attempt at generalizability, nor at having its findings applied to all contexts of CHW supervision. Instead, our study attempts to sensitise readers, including policy makers and implementers, to the need to take note of the experiences of supervisors, which may not be in congruence with their current plans and preparations for CHW interventions. Our findings not only reflect discussions in the literature [2] they also add to this literature. The transferability of the findings from this study will be enhanced when further comparative qualitative studies with supervisors working in an array of different interventions and under an array of different conditions are conducted. It is important to note, nonetheless, that the supervisors' reflections are only one perspective on this intervention. Their perceptions must be compared against those of other intervention participants, as documented in related qualitative sub-studies of this intervention[30].

Although our study is small, it is both credible and trustworthy. We have been transparent in describing our methods, thus bringing attention to the rigour with which we have conducted the study, and opening our methods for peer review. Throughout the study we have engaged in inter-researcher triangulation. The first author in particular has given due consideration to the influence of her subjectivity over the research process and outcomes. We have also considered our findings in relation to the literature, we have validated these findings through participant feedback and peer reporting. While descriptive studies like ours may not offer the highest level of evidence that can be reached from qualitative studies [42], they remain important in areas where there is little other research. Green and Thorogood [43] point out that in researching relatively under-researched topics, the issue of sensitizing readers to new ways of thinking or participant experience is more salient than the issue of generalizability. Given that we have not found any other studies that specifically address the experiences of CHW supervisors, our study is important in sensitising readers to the need to take their experiences into account, and the need to see that supervision can extend well beyond the boundaries of administration, and well into the realm of support. This then is the transferable lesson for researchers and policy makers.

## Conclusions

Supervision is important, not only to CHW interventions but to all of the human resource activities involved in delivering health care. While the literature offers many opinions on the importance of supervision, there is limited evidence and reflection on what actually happens on the ground. This study has shown that there may be a gap between how supervisors are prepared for their task and what they actually do on a daily basis. Future policy making and implementation would be enhanced by more attention to the daily realities of supervisors.

## Competing interests

The authors declare that they have no competing interests.

## Authors' contributions

In this sub-study KD conceptualised the research question in discussion with BN and E-CE as part of the Promise EBF South Africa evaluation. KD conducted the interviews. The transcribed interviews were analysed by KD and BN in discussion with TD. The results of the analysis were discussed and agreed upon by all the authors. KD wrote the first draft of the paper. DJ is a member of international Promise EBF steering committee and was responsible for the South African Study overallm including this sub-study. All authors contributed to and refined subsequent drafts. All authors read and approved the final manuscript.

## Authors' information

DB, E-CE and TD are affiliated with the collaboration, "Promoting Infant Health and Nutrition in Sub–Saharan Africa: Safety and Efficacy of Exclusive Breastfeeding Promotion in the Era of HIV (Promise EBF)".      

## Funding

The community trial is funded by the European Union and the Centre for Disease Control, Atlanta. Additional funding for this qualitative study was provided through a Department of Science and Technology scholarship awarded to Karen Daniels and administered by the Medical Research Council.

## References

[B1] Lewin SA Dick J Pond P Zwarenstein M Aja G van Wyk B Bosch-Capblanch X Patrick M Lay health workers in primary and community health care Cochrane Database Syst Rev 2005 CD004015 15674924 10.1002/14651858.CD004015.pub2

[B2] Lehmann U Sanders D Community health workers: What do we know about them? The state of the evidence on programmes, activities, costs and impact on health outcomes of using community health workers 2007 Geneva: Evidence and Information for Policy, Department of Human Resources for Health, World Health Organisation

[B3] Ofosu-Amaah V National experience in the use of community health workers. A review of current issues and problems WHO Offset Publ 1983 71 1 49 6880399

[B4] Schneider H Hlophe H van Rensburg D Community health workers and the response to HIV/AIDS in South Africa: tensions and prospects Health Policy Plan 2008 23 179 187 10.1093/heapol/czn006 18388133

[B5] Curtale F Siwakoti B Lagrosa C LaRaja M Guerra R Improving skills and utilization of community health volunteers in Nepal Soc Sci Med 1995 40 1117 1125 10.1016/0277-9536(94)00172-P 7597465

[B6] Gilson L Walt G Heggenhougen K Owuor-Omondi L Perera M Ross D Salazar L National community health worker programs: how can they be strengthened? J Public Health Policy 1989 10 518 532 10.2307/3342522 2621254

[B7] Haines A Sanders D Lehmann U Rowe AK Lawn JE Jan S Walker DG Bhutta Z Achieving child survival goals: potential contribution of community health workers Lancet 2007 369 2121 2131 10.1016/S0140-6736(07)60325-0 17586307

[B8] Global Strategy for Infant and Young Child Feeding 2003 World Health Organization

[B9] Dyson L Renfrew M McFadden A McCormick F Herbet G Thomas J Promotion of breastfeeding initiation and duration: Evidence into practice briefing 2005 National Institute for Health and Clinical Excellence

[B10] Clarke M Towards cost-effective tuberculosis control in the Western Cape of South Africa: intervention study involving lay health workers on agricultural farms 2005 Karolinska Institutet, Division of International Health (IHCAR) Department of Public Health Sciences

[B11] Heiby J QAP Editorial: Quality Assurance and Supervision Systems QA Brief: The Quality Assurance Project's Information Outlet 1998 7 1 3 12294098

[B12] Stinson W Malianga L Marquez L Madubuike C Quality Supervision QA Brief: The Quality Assurance Project's Information Outlet 1998 7 4 6 12294100

[B13] Rowe SY Kelly JM Olewe MA Kleinbaum DG McGowan JE Jr McFarland DA Rochat R Deming MS Effect of multiple interventions on community health workers' adherence to clinical guidelines in Siaya district, Kenya Trans R Soc Trop Med Hyg 2007 101 188 202 10.1016/j.trstmh.2006.02.023 17064747

[B14] Doherty T Chopra M Nkonki L Jackson D Greiner T Effect of the HIV epidemic on infant feeding in South Africa: "When they see me coming with the tins they laugh at me" Bull World Health Organ 2006 84 90 96 10.2471/BLT.04.019448 16501725 PMC2626538

[B15] Doherty T Chopra M Nkonki L Jackson D Persson LA A longitudinal qualitative study of infant-feeding decision making and practices among HIV-positive women in South Africa J Nutr 2006 136 2421 2426 16920864 10.1093/jn/136.9.2421

[B16] Doherty TM McCoy D Donohue S Health system constraints to optimal coverage of the prevention of mother-to-child HIV transmission programme in South Africa: lessons from the implementation of the national pilot programme Afr Health Sci 2005 5 213 218 16245991 10.5555/afhs.2005.5.3.213 PMC1831931

[B17] Coutsoudis A Pillay K Kuhn L Spooner E Tsai WY Coovadia HM Method of feeding and transmission of HIV-1 from mothers to children by 15 months of age: prospective cohort study from Durban, South Africa AIDS 2001 15 379 387 10.1097/00002030-200102160-00011 11273218

[B18] Dunn DT Newell ML Ades AE Peckham CS Risk of human immunodeficiency virus type 1 transmission through breastfeeding Lancet 1992 340 585 588 10.1016/0140-6736(92)92115-V 1355163

[B19] Iliff PJ Piwoz EG Tavengwa NV Zunguza CD Marinda ET Nathoo KJ Moulton LH Ward BJ Humphrey JH Early exclusive breastfeeding reduces the risk of postnatal HIV-1 transmission and increases HIV-free survival AIDS 2005 19 699 708 10.1097/01.aids.0000166093.16446.c9 15821396

[B20] Coovadia HM Rollins NC Bland RM Little K Coutsoudis A Bennish ML Newell ML Mother-to-child transmission of HIV-1 infection during exclusive breastfeeding in the first 6 months of life: an intervention cohort study Lancet 2007 369 1107 1116 10.1016/S0140-6736(07)60283-9 17398310

[B21] Thior I Lockman S Smeaton LM Shapiro RL Wester C Heymann SJ Gilbert PB Stevens L Peter T Kim S Breastfeeding plus infant zidovudine prophylaxis for 6 months vs formula feeding plus infant zidovudine for 1 month to reduce mother-to-child HIV transmission in Botswana: a randomized trial: the Mashi Study JAMA 2006 296 794 805 10.1001/jama.296.7.794 16905785

[B22] Agrasada GV Gustafsson J Kylberg E Ewald U Postnatal peer counselling on exclusive breastfeeding of low-birthweight infants: a randomized, controlled trial Acta Paediatr 2005 94 1109 1115 10.1080/08035250510025752 16188857

[B23] Coutinho SB de Lira PI de Carvalho Lima M Ashworth A Comparison of the effect of two systems for the promotion of exclusive breastfeeding Lancet 2005 366 1094 1100 10.1016/S0140-6736(05)67421-1 16182897

[B24] Haider R Ashworth A Kabir I Huttly SR Effect of community-based peer counsellors on exclusive breastfeeding practices in Dhaka, Bangladesh: a randomised controlled trial Lancet 2000 356 1643 1647 10.1016/S0140-6736(00)03159-7 11089824

[B25] Leite AJ Puccini RF Atalah AN Alves Da Cunha AL Machado MT Effectiveness of home-based peer counselling to promote breastfeeding in the northeast of Brazil: a randomized clinical trial Acta Paediatr 2005 94 741 746 10.1080/08035250410023854 16188778

[B26] Morrow AL Guerrero ML Shults J Calva JJ Lutter C Bravo J Ruiz-Palacios G Morrow RC Butterfoss FD Efficacy of home-based peer counselling to promote exclusive breastfeeding: a randomised controlled trial Lancet 1999 353 1226 1231 10.1016/S0140-6736(98)08037-4 10217083

[B27] Chopra M Jackson D Doherty T Colvin M Good Start Community-Based Intervention Study: Ethics Application 2006 Medical Research Council, University of the Western Cape & Centre for AIDS Development Research and Evaluation

[B28] Tylleskär T Chopra M Diallo H Doherty T Ekström E-C Engebretsen IM Goga A Jackson D Kankasa C Klungsøyr J PROMISE Final Scientific Report:- Preliminary results from the multi-centre cluster-randomised behaviour intervention trial PROMISE EBF: exclusive breastfeeding promotion in Sub-Saharan Africa 2009 University of Bergen

[B29] Chopra M Doherty T Jackson D Ashworth A Preventing HIV transmission to children: quality of counselling of mothers in South Africa Acta Paediatr 2005 94 357 363 16028656 10.1111/j.1651-2227.2005.tb03080.x

[B30] Nor B Zembe Y Daniels K Doherty T Jackson D Ahlberg BM Ekstrom EC "Peer but Not Peer": Considering the Context of Infant Feeding Peer Counseling in a High HIV Prevalence Area J Hum Lact 2009 19622755 10.1177/0890334409341050

[B31] Goga A *Personal Communication* 2009

[B32] Mind the gap: equity and trends in coverage of maternal, newborn, and child health services in 54 Countdown countries Lancet 371 1259 1267 10.1016/S0140-6736(08)60560-7 18406860

[B33] Lewin S Bridging the equity gap in maternal and child health: lay health workers may help bridge equity gap in maternal and child health BMJ 2005 331 844 10.1136/bmj.331.7520.844-a 16210301 PMC1246130

[B34] Cattaneo A Promoting breast feeding in the community BMJ 2009 338 a2657 10.1136/bmj.a2657 19181728

[B35] Hoddinott P Britten J Prescott GJ Tappin D Ludbrook A Godden DJ Effectiveness of policy to provide breastfeeding groups (BIG) for pregnant and breastfeeding mothers in primary care: cluster randomised controlled trial BMJ 2009 338 a3026 10.1136/bmj.a3026 19181729 PMC2635594

[B36] MacArthur C Jolly K Ingram L Freemantle N Dennis CL Hamburger R Brown J Chambers J Khan K Antenatal peer support workers and initiation of breast feeding: cluster randomised controlled trial BMJ 2009 338 b131 10.1136/bmj.b131 19181730 PMC2636685

[B37] Department of Health SA Community Health Worker Policy Framework Pretoria 2005

[B38] Moran VH Dykes F Burt S Shuck C Breastfeeding support for adolescent mothers: similarities and differences in the approach of midwives and qualified breastfeeding supporters Int Breastfeed J 2006 1 23 10.1186/1746-4358-1-23 17125521 PMC1687180

[B39] Sarafino EP Health Psychology: Biospsychosocial Interactions 1994 New York: John Wiley & Sons

[B40] Sarafino EP Health Psychology: Biopsychosocial Interactions 2005 5 New York: John Wiley & Sons

[B41] Marais H A plague of inequality Mail &Guardian 2006

[B42] Daly J Willis K Small R Green J Welch N Kealy M Hughes E A hierarchy of evidence for assessing qualitative health research J Clin Epidemiol 2007 60 43 49 10.1016/j.jclinepi.2006.03.014 17161753

[B43] Green J Thorogood N Qualitative Methods for Health Research 2004 London: Sage Publications

